# The Administration of Anesthetics

**Published:** 1895-04

**Authors:** Jas. B. Morgan

**Affiliations:** Augusta, Ga.


### THE ADMINISTRATION OF ANESTHETICS.*

By JAS. B. MORGAN, M.D.,

AUGUSTA, GA.

In this paper I call attention to the great dangers attendant upon the improper use of these agents. Doctors are often unwilling to report fatal cases occurring in their practice, and hence the mortality from ether and chloroform is really greater than is generally supposed. At a recent meeting of the New York Academy of Medicine, where a paper on Anesthetics, by Dr. Gerster, was under discussion, several cases of death from this cause were narrated by different members that had never been reported. Correct statistics cannot be obtained until all public hospitals register and publish the results of all cases of surgical anesthesia. In the hands of different surgeons the death-rate varies greatly. Thus, Surgeon Major Lawrie, of the English army, has seen chloroform given over 50,000 times without a death, and Hunter McGuire has used it 28,000 times without a single fatal result; Chisholm, of Baltimore, has had no accident in some 30,000 cases. On the other hand, the use of chloroform in the medical service of the United States army has been fatal once in every 2,000 cases, and Anstie lost one case in every 145. The mortality from ether is placed at from 1 in 10,000 to 1 in 80,000. This difference in death-rate grows out of difference in the use of anesthetics. The practice of intrusting the youngest doctor at an operation with the important duty of anesthetizing the patient is fraught with the greatest danger and should be most earnestly condemned. There is great variety of opinion as to the choice of an anesthetic. Each agent having strong advocates who, in their zeal, often degenerate into blind partisans.

In the United States ether is the drug most employed, while in England and Germany the preference is for chloroform. The truth

*Abstract of a paper read at the Augusta Academy of Medicine, February 6, 1895.

is, that each agent is useful in its proper sphere. One being best suited to a certain class of cases, and the other to another class of cases. According to the statistics of Julliard of Geneva, which are the most complete on this subject, ether mortality is 1 in 14,987 cases, while that of chloroform is 1 in 4,301. The condition of the patient must indicate the anesthetic to be employed; thus ether is contraindicated in cases of nephritis, chronic bronchitis, and in those persons who resist its anesthetic effect. Chloroform is best for young children, because of their narrow air passages and for operations which become necessary immediately after the patient has eaten a hearty meal. It is also the best anesthetic for obstetric use. When the operation is upon the respiratory organs chloroform is much better than ether. Before a patient is anesthetized a thorough examination should always be made in order that the surgeon should know and thus be able to guard against the weak points. The heart, lungs, and kidneys should be especially investigated, because it is in this direction that the danger lies. A weak, irregular pulse is not necessarily a positive contraindication, because often under the stimulant effect of ether the heart will become steady and strong in its action. The best preventive of accidents is to thoroughly prepare the patient for the anesthetic beforehand. This preparatory treatment should often be begun weeks or even months before the operation. The weak and debilitated patient should be built up with tonics and stimulants. Often a course of strychnia and digitalis is of service in strengthening the heart. The presence of albumin in the urine also most urgently calls for preliminary treatment. Before the anesthetic is given the stomach should be thoroughly emptied by some simple, rapidly acting emetic. The patient must always be upon the back, with the clothes perfectly loose about the chest and abdomen, and should always be given sulphate of morphia and atropia before inhaling the drug. This last precaution is especially benefical when chloroform is used, for, according to Aubert, a distinguished French surgeon, it materially adds to the safety of the anesthetic, aids greatly in the rapid induction of narcosis, diminishes the amount of anesthetic used, prevents vomiting, and favors quick return to consciousness. It is an error to suppose that in the administration of ether

545 - May 12 [illegible]

and chloroform, that with the first drug an admixture of air should not be allowed, while it is necessary with the second. Anesthesia is produced not by asphyxia, but by the direct action of the agent used upon the nerve centers. Any method, therefore, that deprives the patient of oxygen, substitutes the unconsciousness of suffocation for true anesthesia. The necessity for air exists, and is just as great with ether as with chloroform.

Fatalities always occur early in the administration; hence do not crowd the anesthetic at first, but begin slowly and allow the patient plenty of fresh air. The reasons why accidents almost always occur during the first stage of anesthesia is because the drug is drawn into the lungs in too concentrated a form; thus, chloroform paralyzes the heart, and ether, by sudden evaporation, chills the tissues and irritates the bronchial tubes and fauces, thus producing spasm of the glottis, reflex cough, profuse secretion of mucus, and, secondarily, pneümonia. For chloroform, the Esmarch mask and dropper is the best form of inhaler for general use, though the Junker is a more perfect instrument. For ether, the Allis inhaler subserves the best purpose, as it is simple and allows plenty of air. The Clover inhaler is a most expensive and dangerous machine, and should not be used, as it requires the patient to breathe over and over again the same air, and thus produces suffocation rather anesthesia. It is very important that the anesthetic should be pure. The ether and chloroform manufactured by Squibb is generally regarded by the profession as of superior quality. The most important danger signal in the use of anesthetics is the condition of the pupil, for it accurately indicates when the cerebrum is completely narcotized, and is much more reliable than the pulse or respiration. The pupil passes through certain regular changes as the patient goes under the drug. It is at first dilated and active, then becomes closely contracted, and, lastly, widely dilated and fixed. The first state is the sign of imperfect anesthesia, the last indicates too much narcosis; hence the golden mean is shown by a contracted pupil. Whenever the pupil begins to dilate, the anesthetizer should look out, for the patient is either coming round and beginning to develop reflexes, or dangerous action upon the respiratory and cardiac centers is about to supervene. It is easy to diagnosticate between commencing reflex and early narcotic dilatation, for in the first condition the pupil is active, the respiration shallow, and vomiting and other movements occur; while in the latter the breathing is stertorous, the eyeballs fixed, and the conjunctiva is not sensitive. In the first state, the indication is for more of the anesthetic; in the second, its administration should at once be suspended until contraction of the pupil occurs. If the eye be read in this way, all interference with respiration and the heart's action will be avoided. The sign applies with equal force whether chloroform or ether is the anesthetic.

[In the discussion which followed the reading of this paper, Dr. Jos. Eve Allen said that the mortality statistics of anesthetics were at present very unreliable owing, as the essayist of the evening had very properly pointed out, to the reluctance of medical men to report their fatal cases. He had had, in his own practice, a death from chloroform that had never been reported. It occurred in the case of an old man upon whom the operation for strangulated hernia was about to be performed. In this instance the heart was paralyzed just as the inhalation was begun. The two most important points brought out by the paper just read were: first, that the admixture of air is just as necessary when ether is used as when chloroform is the anesthetic, a fact which is not generally known and recognized, and to which may be attributed the majority of ether accidents and, second, the great value of the pupil as a danger signal.

Chloroform is the agent best suited to obstetric practice, because it is more manageable than ether, does not produce nausea and vomiting, can be used at night without the risk of explosion, and is more uniform in its effects. Dr. Allen had used chloroform in over 3,000 cases of labor without a single bad result. The safety of this drug in pregnant and parturient cases is due to the physiological increase in the heart muscle, especially of the left ventricle, that takes place during gestation. The use of chloroform during labor is, however, not entirely without danger; for, if pushed too far, its secondary effects seriously complicate the third stage by producing irregular uterine action or complete relaxation; and retention of the placenta, with hour-glass contraction or excessive post-partum hemorrhage, is the result. The use of chloroform should be restricted in normal labor cases to the second stage. It is to be given only during the pains, the inhalation beginning with the deep inspiration that the woman always involuntarily takes preparatory to the expulsive efforts, and stopping before the contraction has entirely ceased. Anesthesia should never be carried to the surgical extent unless some obstetric operation is to be performed.

Ether is theoretically the best anesthetic for diagnostic examinations for elective operations, such as the induction of premature labor, the Cæsarean section, and symphyseotomy, and for use during the third stage, when removal of the placenta is necessary or perineal lacerations are to be repaired. In the great majority of these cases, however, chloroform can be employed with equally good effect. Ether cannot be used when renal disease is present, and hence chloroform is the drug to be used in puerperal eclampsia. Profound and long-continued anesthesia of the mother often has an injurious effect upon the fetus *in utero*, and in many cases the occurrence of still-births may be traced to this cause.]

